# The oncometabolite D-2-hydroxyglutarate induced by mutant IDH1 or -2 blocks osteoblast differentiation *in vitro* and *in vivo*

**DOI:** 10.18632/oncotarget.4024

**Published:** 2015-05-25

**Authors:** Johnny Suijker, Hans J. Baelde, Helene Roelofs, Anne-Marie Cleton-Jansen, Judith V.M.G. Bovée

**Affiliations:** ^1^ Department of Pathology, Leiden University Medical Center, Leiden, The Netherlands; ^2^ Department of Immuno-Haematology and Blood Transfusion, Leiden University Medical Center, Leiden, The Netherlands

**Keywords:** isocitrate dehydrogenase, enchondroma, mesenchymal stem cells, ollier disease, maffucci syndrome

## Abstract

Mutations in *isocitrate dehydrogenase 1* (*IDH1*) and *IDH2* are found in a somatic mosaic fashion in patients with multiple enchondromas. Enchondromas are benign cartilaginous tumors arising in the medulla of bone. The mutant IDH1/2 causes elevated levels of D-2-hydroxyglutarate (D-2-HG). Mesenchymal stem cells (MSC) are the precursor of the osteoblastic, chondrogenic and adipocytic lineage and we hypothesized that increased levels of D-2-HG cause multiple enchondromas by affecting differentiation of MSCs. Bone marrow derived MSCs from different donors were differentiated towards osteoblastic, chondrogenic and adipocytic lineage in the presence or absence of 5 mM D-2-HG. Three of four MSCs showed near complete inhibition of calcification after 3 weeks under osteogenic differentiation conditions in the presence of D-2-HG, indicating a block in osteogenic differentiation. Two of four MSCs showed an increase in differentiation towards the chondrogenic lineage. To evaluate the effect of D-2-HG *in vivo* we monitored bone development in zebrafish, which revealed an impaired development of vertebrate rings in the presence of D-2-HG compared to control conditions (*p*-value < 0.0001). Our data indicate that increased levels of D-2-HG promote chondrogenic over osteogenic differentiation. Thus, mutations in *IDH1/2* lead to a local block in osteogenic differentiation during skeletogenesis causing the development of benign cartilaginous tumors.

## INTRODUCTION

Enchondroma is a benign cartilage forming tumor within the medullary cavity of the bone [1, 2]. Enchondromas can occur as solitary lesions, of which the exact incidence is unknown as they are often detected when radiographs are made for other reasons. Multiple enchondromas are seen in patients with the rare enchondromatosis syndrome. Different subtypes of enchondromatosis are distinguished, of which the most common ones are Ollier disease and Maffucci syndrome, the latter distinguished by multiple spindle cell hemangiomas in addition to enchondromas [2, 3]. We and others have shown that both these non-hereditary syndromes are caused by somatic mosaic heterozygous mutations in the *isocitrate dehydrogenase 1* (*IDH1*) or *isocitrate dehydrogenase 2* (*IDH2*) genes [4–7]. Up to 87% of solitary or multiple enchondromas harbor mutations in *IDH1* or *IDH2* [4–6].

Malignant progression towards secondary central chondrosarcoma occurs in ~1% of solitary enchondromas and up to ~50% in patients with multiple enchondromas [8]. Central chondrosarcomas are malignant cartilage-forming tumors that are located in the medulla of the bone [9]. Central conventional chondrosarcomas carry mutations in *IDH1* or *IDH2* in 38–70% of primary central chondrosarcomas (arising without a preexisting benign enchondroma) and in 86% of the secondary central chondrosarcomas [4–6].

Mutations in *IDH1* or *IDH2* are also found in acute myeloid leukemia (~20%) [10], gliomas (60–80%) [11, 12], and cholangiocarcinomas (7–28%) [13–15]. Isocitrate dehydrogenase is a metabolic enzyme that catalyzes the conversion of isocitrate to α-ketoglutarate in the TCA cycle. Mutant IDH1/2 has been proven to have neo-activity for the catalysis of α-ketoglutarate into D-2-hydroxyglutarate (D-2-HG), but not to its enantiomer L-2-hydroxyglutarate (L-2-HG) [16, 17]. Indeed, enchondromas and other tumors with mutations in *IDH*1 or *IDH*2 were shown to have increased levels of D-2-HG [4]. The newly formed oncometabolite D-2-HG shows structural similarities with α-ketoglutarate, and as a result D-2-HG is able to competitively inhibit α-ketoglutarate dependent enzymes, such as TET2, thereby inducing epigenetic changes including DNA hypermethylation and histone modification [18]. Epigenetic changes due to mutant IDH protein were demonstrated to impair hematopoietic differentiation of hematopoietic precursor and leukemia cells [19–21] and to repress neural differentiation of neurogenic precursor cells [22]. Moreover, mutant IDH and D-2-HG prevent liver progenitor cells from undergoing hepatocyte differentiation, causing intrahepatic cholangiocarcinoma [23].

As the mesenchymal stem cell (MSC) is the precursor of the osteogenic, chondrogenic and adipocytic lineage, we hypothesized that enchondromas and chondrosarcomas result from altered differentiation of MSCs during bone development. Our aim was therefore to investigate whether *IDH1/2* mutations and the corresponding elevated levels of D-2-HG push the differentiation of MSCs towards a more chondrogenic phenotype by blocking osteogenic differentiation. We therefore used human MSCs that were pushed towards osteogenic, chondrogenic and adipocytic differentiation in the presence or absence of D-2-HG, as a surrogate for the *IDH1* or *-2* mutation. In addition, a zebrafish model was used to evaluate the effect of D-2-HG on bone development *in vivo*.

## RESULTS

### D-2-HG inhibits osteogenic differentiation of MSCs

**Figure 1:**
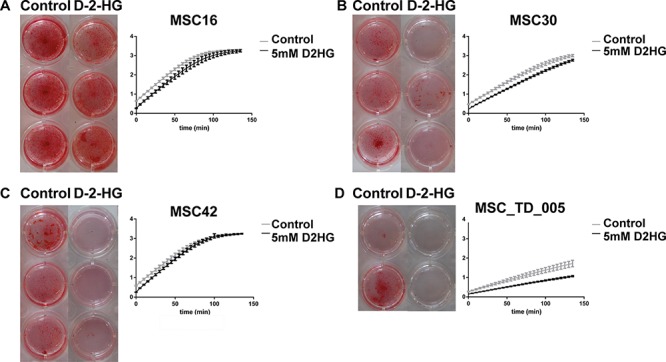
Osteogenic differentiation of mesenchymal stem cells in the presence of D-2-HG **A–D:** For MSCs of four different donors (A-D) alizarin red staining (left panel) and alkaline phosphatase activity (right panel) are shown after 3 weeks of osteoblast differentiation. Alizarin Red staining for osteogenic differentiation was performed in triplicate (A-C) or duplicate (not enough cells to perform triplicate) (D) in the presence of 5 mM D-2-HG or in its absence (−). MSCs showed calcification after 3 weeks in control conditions, while three out of four MSCs showed near complete inhibition of calcification in the presence of D-2-HG. The right panels demonstrate alkaline phosphatase activity measured every 5 minutes for 140 minutes, representing six different measurements per MSC strain. At three weeks, MSC_TD_005 (D) clearly demonstrated reduced alkaline phosphatase activity in the presence of D-2-HG (slope of 0.0538 (control) compared to 0.0341 (in the presence of D-2-HG)).

MSCs that were pushed towards osteogenic differentiation in the presence of 5 mM D-2-HG, as a surrogate for *IDH1* or *-2* mutation, showed impaired calcification after three weeks as compared to MSCs pushed towards osteogenic differentiation in the absence of D-2-HG. More specifically, 3 out of 4 MSC strains (MSC30, MSC42 and MSC_TD_005) showed near complete inhibition of calcification in the presence of D-2-HG, whereas in MSC16 alizarin red staining was not affected (Fig. 1). The MSCs of a fifth donor failed to show any osteoblast differentiation in control conditions. Alkaline phosphatase activity was determined after three weeks of differentiation by measuring at time intervals of 5 minutes during 140 minutes. This clearly demonstrated reduced alkaline phosphatase activity in MSC_TD_005 (Fig. 1D) in the presence of D-2-HG (slope of 0.0538 (control) compared to 0.0341 (in the presence of D-2-HG)). In the other MSC strains, no difference in the slopes of the curves could be detected when comparing activity between control conditions and D-2-HG.

### Effect of D-2-HG on chondrogenic differentiation of MSCs is variable

**Figure 2:**
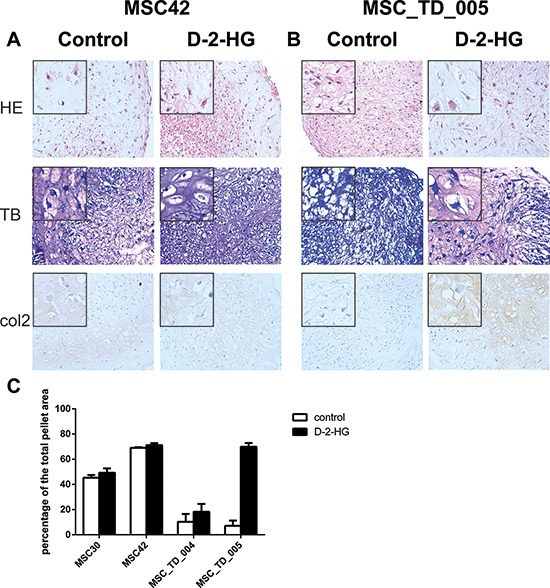
Chondrogenic differentiation of mesenchymal stem cells in the presence of D-2-HG Pellets were grown, in duplicate, in chondrogenic differentiation medium in the presence and absence of D-2-HG for four weeks and results were variable. **A.** Representative stainings (MSC42) for pellets without an effect on differentiation after D-2-HG treatment. **B.** Representative stainings for pellets (MSC_TD_005) that showed increased chondrogenic differentiation after treatment with D-2-HG. **C.** Quantification of metachromasia in toluidine blue staining after chondrogenic differentiation. Results from MSCs from 4 different donors are shown in percentages of the total pellet area.

The effect of D-2-HG on chondrogenic differentiation, evaluated after four weeks, was variable in four donors when comparing pellets grown in the presence or absence of D-2-HG. MSCs of a fifth donor were discarded due to an infection. All pellets revealed some level of chondrogenic differentiation on haematoxylin and eosin staining and all pellets were positive using immunohistochemistry for collagen II, and negative for collagen X. Morphologically, in two out of four samples (MSC_TD_004 and MSC_TD_005) matrix deposition as well as collagen II staining were increased when grown in the presence of D-2-HG as compared to pellets grown in the absence of D-2-HG (Fig. 2B). Moreover, an increase in chondrogenic differentiation in the presence of D-2-HG as defined using quantification of metachromasia at toluidine blue staining was confirmed in MSC_TD_004 and MSC_TD_005 (Fig. 2C, Supplementary Fig 1). In MSC42 there was only a slight increase in metachromasia while in MSC30 an increase in chondrogenic differentiation in the presence of D-2-HG could not be detected.

### D-2-HG does not have a detectable effect on histone modifications and DNA methylation in MSCs after chondrogenic differentiation

As D-2-HG was reported to affect histone modification and DNA methylation we evaluated histone marks and DNA methylation using immunohistochemistry. No difference in trimethylation of H3K4, H3K9 and H3K27 could be detected in pellets grown in the presence of D-2-HG as compared to pellets grown under normal control conditions. We demonstrated high levels of the histone modification marks H3K4me3 and H3K27me3, whereas staining for H3K9me3 was negative in both conditions. Furthermore, 5-mC as well as 5-hmC was highly expressed in pellets grown in control conditions as well as in those grown in the presence of D-2-HG. Results are shown in Supplementary Figure 2.

### D-2-hydroxyglutarate does not influence adipocyte differentiation

**Figure 3:**
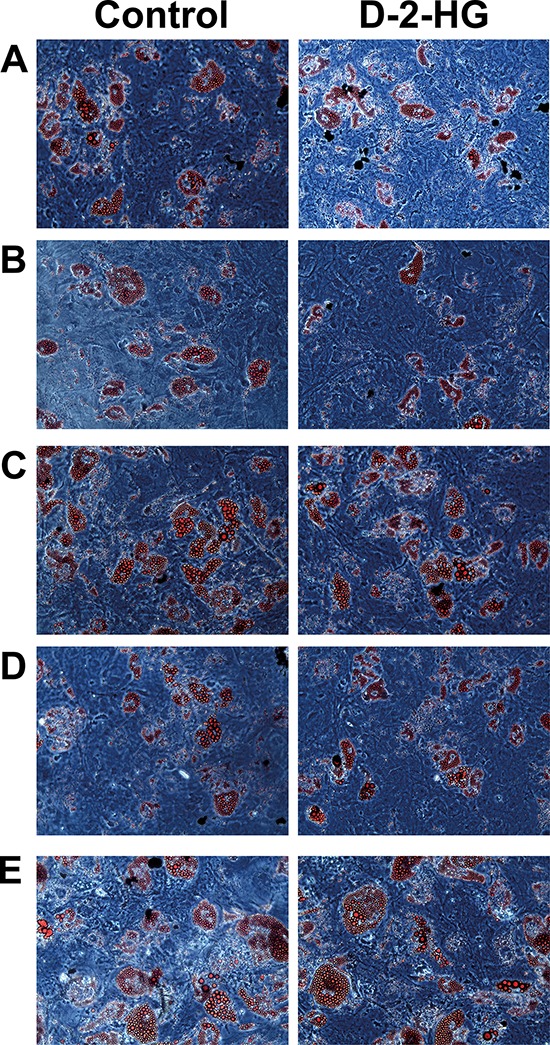
Oil-red-O staining of 5 MSCs to determine the effect of D-2-HG on the ability to differentiate into adipocytes Left panel shows the ability of MSCs from 5 different donors to differentiate into adipocytes. The right panel shows the differentiation after treatment with 5 mM D-2-HG. **A.** MSC16, **B.** MSC30, **C.** MSC42, **D.** MSC_TD_004 and **E.** MSC_TD_005 were all able to differentiate into adipocytes after treatment with D-2-HG. No difference could be detected between treated and untreated samples.

As a control, we also evaluated the effect of D-2-HG on adipocytic differentiation. All five MSC strains pushed towards adipocytic differentiation for 3 weeks were able to differentiate as demonstrated by the formation of lipid vesicles using Oil red O staining. There was no difference in adipocytic differentiation between MSCs grown in the presence of 5 mM D-2-HG or PBS (Figure 3). Further evidence for the lack of influence of D-2-HG in adipocyte differentiation was demonstrated in MSCs that were pushed towards osteogenic differentiation for 3 weeks, where spontaneous adipocyte differentiation was seen both in the presence and absence of 5 mM D-2-HG (Supplementary Fig. 3).

### D-2-HG impairs bone development in zebrafish embryos

**Figure 4:**
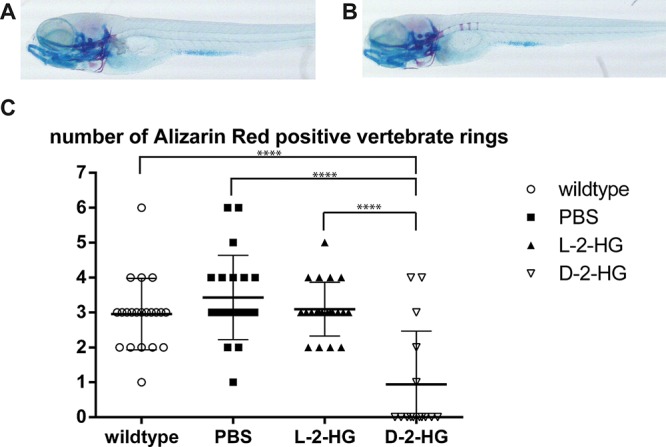
Bone development in zebrafish embryos in the presence of D-2-HG Representative photographs of zebrafish embryos eight days after fertilization, visualized by double staining of alcian blue (staining cartilaginous elements) and alizarin red (staining osteogenic elements). **A–B:** When zebrafish embryos developed in the presence of D-2-HG (A) no alizarin red positive vertebrate rings are detected when compared to zebrafish grown in normal control conditions (B) displaying three alizarin red positive vertebrate rings **C:** Quantification of vertebrate rings in all zebrafish revealed an average of three alizarin red positive vertebrate rings in the control conditions (ranging from 2.95 (without injection) to 3.42 (injected with PBS)). Zebrafish that were injected with D-2-HG in their yolk sac showed a significantly reduced number of vertebrate rings (average 0.93), indicating that the presence of D-2-HG impairs bone development in zebrafish embryos. The numbers represent one independent experiment. ****p < 0.0001.

A zebrafish model was used to investigate the effect of D-2-HG on bone development *in vivo*. The number of alizarin red positive vertebrate rings, as a measurement for development of the bony skeleton, at eight days post fertilization (dpf), significantly differed between zebrafish embryos with and without injection of D-2-HG at day 0. Overall, the zebrafish embryos injected with D-2-HG mainly showed zebrafish embryos lacking alizarin red positive vertebrate rings while zebrafish embryos that developed in the control conditions (no injection, PBS or injection with the enantiomer L-2-HG) on average revealed three vertebrate rings at 8 dpf. More specifically, embryos without injection demonstrated an average of 2.95 alizarin red positive vertebrate rings (*n* = 21). Embryos injected with PBS had an average of 3.42 alizarin red positive vertebrate rings (*n* = 21). Injection with the enantiomer L-2-HG resulted in an average number of 3.1 vertebrate rings (*n* = 21). In contrast, injections with D-2-HG resulted in significantly less alizarin red positive rings with an average of 0.93 vertebrate rings (*n* = 16) (*p*-value < 0.0001), implicating impaired bone development (Fig. 4, Supplementary Fig. 4). Experiments with PBS and D-2-HG were performed twice at different time points, and results were comparable. When evaluating the alcian blue staining visually, no difference between zebrafish embryos injected with D-2-HG, L-2-HG or PBS was seen.

## DISCUSSION

We here demonstrate that elevated levels of D-2-HG block osteogenic differentiation and variably promote chondrogenic differentiation of mesenchymal stem cells. Elevated levels of D-2-HG are caused by mutations in *IDH*1 or *IDH*2, which are frequently found (up to 87%) in enchondromas. It is therefore likely that a local block in osteogenic differentiation causes the cartilaginous mass (enchondroma) during bone development. In solitary enchondromas, the mass may be detected later during life, when X-rays are made for other reasons, or when enchondromas progress towards secondary central chondrosarcoma. In multiple enchondroma syndromes, the somatic mosaic distribution of the *IDH*1 or *IDH*2 mutation in the MSCs during bone development underlies the development of these lesions. The amount of deformity of the skeleton depends on the mutational load since the amount of cartilaginous masses in these patients is variable.

We convincingly showed both *in vitro* as well as *in vivo* that osteogenic differentiation is inhibited. Three out of four MSCs showed a near complete inhibition of calcification in the presence of D-2-HG, and zebrafish embryos injected with D-2-HG at day 0, failed to develop vertebrate rings when examined at day 8, whereas alkaline phosphatase activity was reduced in one out of four MSCs. The discrepancy between the alizarin red staining and the alkaline phosphatase activity could be explained by the fact that alkaline phosphatase activation occurs in earlier phase of osteogenesis than osteogenic matrix production [24].

The mechanism of tumorigenesis is therefore comparable to other tumors caused by mutations in the *IDH1* or *IDH2* gene, as both in hematopoietic precursor cells, in neurogenic precursor cells as well as in liver progenitor cells, differentiation was impaired. This is contributing to the development of leukemia, glioma and cholangiocarcinoma, respectively, all of which are described to have *IDH1* or *-2* mutations in a relatively high percentage of tumors [19–23].

Interestingly, we here show that D-2-HG blocks osteogenic differentiation, while mutations are virtually absent in osteosarcoma and very frequent in cartilage tumors [4]. The effect of D-2-HG on chondrogenic differentiation of MSCs was variable, and seemed to promote chondrogenic differentiation in only half of the MSCs. The variable effect of D-2-HG on chondrogenic differentiation of MSCs is not surprising, as we and others have previously described large intra- and inter-donor variability of MSCs [25–27], which was the reason for including MSCs of multiple donors in our study. In the zebrafish embryos, no effect on the development of the cartilaginous elements of the skeleton was seen at day 8. Given the pluripotency of the MSC, which is thought to be the progenitor cell of many different types of sarcomas, our data indicate that elevated levels of D-2-HG, by blocking osteogenic differentiation, enable or even promote chondrogenic differentiation. Very recently, and in line with our results, Hirata et al. reported dysregulated chondrogenic differentiation with persistence of hypertrophic chondrocytes in *Col2a1*-Cre;*IDH1*-KI mice, preventing the normal replacement of cartilage by bone [28].

In addition to osteogenic and chondroblastic differentiation, MSCs can also differentiate towards adipocytes. We here show that D-2-HG does not affect adipocytic differentiation of MSCs. This is in contrast to the reduced adipogenic differentiation caused by D2HG or by introduction of an *IDH2* mutation in immortalized embryo-derived 3T3-L1 cells [22]. These murine 3T3-L1 cells are known to be committed to spontaneous adipogenic differentiation, while we studied the effect on human MSCs, which may explain the different results. Furthermore, we also observed spontaneous adipocyte differentiation in MSCs treated with D-2-HG under osteogenic differentiation conditions, indicating a lack of influence of D-2-HG on the ability of human MSCs to differentiate into adipocytes (Supplementary Fig. 3).

We used D-2-HG as a surrogate for mutations in *IDH1* or *-2*. Elevated levels of the oncometabolite D-2-HG competitively inhibit α-ketoglutarate dependent enzymes, such as TET2 [16]. TET2 normally alters the epigenetic status of DNA by oxidizing 5-methylcytosine (5 mC) to 5-hydroxymethylcytosine (5 hmC). Indeed, we previously showed hypermethylation in *IDH1* mutant enchondromas [6]. D-2-HG also inhibits other α-ketoglutarate dependent oxygenases [29, 30] such as the Jumonji domain histone demethylases, thereby increasing histone methylation as well [22]. More specifically, trimethylation of H3K4, H3K9 and H3K27 was increased [18, 20, 22]. We evaluated the expression of 5 mC, 5 hmC, H3K4me3, H3K9me3 and H3K27me3 in MSCs after 4 weeks of chondrogenic differentiation in the presence or absence of D-2-HG and could not detect any differences in expression. This could suggest that the effect of D-2-HG on MSCs is independent of DNA methylation or histone modification. However, we feel this is unlikely as we cannot rule out that changes occurred at an earlier time point before terminal differentiation. Also, we were only able to perform immunohistochemistry on the MSCs after chondrogenic differentiation, while D-2-HG predominantly affected MSCs that were pushed towards osteoblastic differentiation. Moreover, immunohistochemistry is only semi quantitative and will probably not detect minor changes. Also, neural cells infected with mutant *IDH1* showed increased H3K9me3 levels only at passage 12, while changes in other methylation marks were delayed and less prominent [22]. As MSCs are slowly proliferating cells with a limited lifespan it is not possible to acquire so many passages, or to acquire sufficient cells for western blot analysis.

While we demonstrate an effect for D-2-HG on bone development, this effect was absent for the enantiomer L-2-HG. Patients with the organic acidurias D-2-hydroxyglutaric aciduria (D-2-HGA) or L-2-hydroxyglutaric aciduria (L-2-HGA) accumulate D-2-HG or L-2-HG in urine, plasma and CSF, causing neurological impairment at young age [31]. D-2-HGA is caused by mutations in either the *D2HGDH* gene, encoding D-2-hydroxyglutarate dehydrogenase (D-2-HGDH), or specific gain-of-function mutations in the *IDH2* gene [32]. Interestingly, multiple patients with D-2-HGA have been described with multiple enchondromas in the metaphysis of the long bones combined with dysplastic vertebral bodies (spondyloenchondromatosis) [33–35]. These aberrations are not described for patients with L-2-HGA. This is completely in line with our results in zebrafish, in which only D-2-HG and not L-2-HG impairs bone development.

Our results are in line with the hypothesis that mutations in *IDH1* or *-2* are early events in the development of cartilaginous tumors. We previously reported that malignant progression towards central chondrosarcoma renders chondrosarcoma growth independent of these mutations. Inhibitors of mutant IDH1 and −2 are currently in development and in clinical trial [36–38]. However, the use of such a mutant IDH1 inhibitor (AGI-5198) decreased D-2-HG levels in a dose dependent manner in three chondrosarcoma cell lines with endogenous *IDH1* mutations, whereas proliferation and migration were not affected [39]. Furthermore, global gene expression, CpG island methylation as well as histone H3K4, −9, −27 trimethylation levels remained unchanged after 20 passages of continuous treatment with AGI-5198 [39]. Thus, while mutations in *IDH1* or -*2* are not essential for chondrosarcoma proliferation, the results of the present study show an effect of D-2-HG on differentiation of MSCs during bone development, promoting chondrogenic and inhibiting osteogenic differentiation, indicating a crucial role for *IDH1/2* mutations in the development of benign enchondromas.

## MATERIALS AND METHODS

### Cell culture

Bone marrow derived human multipotent mesenchymal stem cells of in total five healthy donors(MSCs) were cultured in low glucose DMEM (22320-022; Gibco, Invitrogen Life-Technologies) supplemented with 10% heat-inactivated fetal bovine serum (FBS) (758073; Greiner Bio One) and 1% penicillin/streptomycin (P/S) (100 U/ml). Cells were grown at 37°C in a humidified incubator with 95% air and 5% CO2. Phenotyping of MSCs was done as described previously using FACS analysis [40]. Written informed consent was obtained from all donors prior to bone marrow harvesting according to the procedures as accorded by the LUMC ethical board (protocol number P11.089). All samples were handled in a coded (anonymized) fashion.

### Compounds

D-2-hydroxyglutarate (RC402; PepTech Corporation) and L-2-hydroxyglutarate (90790; Sigma-Aldrich) were dissolved in PBS.

### Osteoblast and adipocyte differentiation

Cells were seeded at a density of 15 000 cells/cm2 in 0.1% gelatin coated plates. Culture medium used was α-MEM supplemented with 10% FCS, 1% glutamax and 1% P/S. For adipocyte differentiation, medium was additionally supplemented with 0.25 μM dexamethasone (Sigma-Aldrich), 50 μg/ml ascorbic acid (Sigma-Aldrich), insulin 100 μg/ml (Sigma-Aldrich), 0.5 mM 1-methyl-3-isobutylxantine (IBMX) (Sigma-Aldrich) and after 4 days of culture 50 μM indomethacin (Sigma-Aldrich) was added. For osteoblast differentiation, medium was additionally supplemented with 50 μg/ml ascorbic acid, 0.1 μM dexamethasone and after 7 days of culture 5 mM β-glycerolphosphate was added. D-2-HG treatment, with a concentration of 5 mM, was started since day 1. Concentration was equivalent to the concentrations measured in gliomas with an *IDH1* mutation. [16, 18] Medium was refreshed twice a week and after 3 weeks of culture, cells were fixed for 10 minutes with 4% paraformaldehyde and stained with Oil-Red-O (adipocyte differentiation) or stained with alizarin red (osteoblast differentiation).

### Alkaline phosphatase assay

Medium was removed and cells were washed twice with PBS. The cells were lysed in ALP lysis buffer (10 mM glycine; 0.1 mM MgCl2; 0.01 mM ZnCl2; pH 10.5; 0.1% Triton X-100) for 3 hours. The assay was performed with 25 μl per sample with 2 μl of ALP substrate (6 mM p-nitrophenylphosphate, PNPP) and 198 μl of ALP assay buffer (100 mM glycine; 1.0 mM MgCl2; 0.1 mM ZnCl2; pH 10.5). ALP activity was directly measured at 405 nm with a Victor3V, 1420 Multilabel plate reader (Perkin Elmer, NL) for 120 minutes, with measurements every 5 minutes. The slopes of the curves were compared between controls and samples treated with 5 mM D-2-HG.

### Chondrogenic differentiation

Cells were seeded in a 96-U-shaped-well plate at a density of 200 000 cells per well and pellets were made by centrifuging the plate at 1200 rpm for 7 minutes. Cells were cultured in DMEM high glucose (Invitrogen), 1% P/S, 1% glutamax, 40 μg/ml proline (Sigma-Aldrich), 100 μg/ml sodium pyruvate (Sigma-Aldrich), 10 μg/ml ITS+premix (Becton Dickinson), 50 μg/ml ascorbic acid, 0.1 μM dexamethasone, 10 ng/ml TGF-β3 (R&D Systems) and 500 ng/ml BMP-6 (R&D Systems). D-2-HG treatment was started since day 1. Cells were allowed to differentiate for 4 weeks. After harvesting, the cells were washed with PBS and fixed with 4% paraformaldehyde, covered by Cytoblock (Shandon Cytoblock, Thermo Scientific, Etten-Leur, NL) and embedded in paraffin. Subsequently, sections were cut, the middle of the pellets was determined, and sections were mounted onto APES-coated slides. Pellet morphology and matrix were examined by hematoxylin and eosin as well as toluidine blue staining. To quantify chondrogenic differentiation, metachromasia, as a measure for the deposition of glycosaminoglycans in the cartilaginous matrix, was measured with the multispectral imaging system (Nuance FX, Cambridge Research & Instrumentation, Inc [CRi], Woburn, MA) on the microscope (Leica DM4000B). This system enabled us to spectral unmix different wavelengths (range, 420–720 nm) and thereby distinguish the purple and blue staining. After subtracting the different colors, percentages of collagen positive areas were determined using the imageJ software (U.S. National Institutes of Health, Bethesda, MD).

### Immunohistochemistry

Immunohistochemistry was performed for histone modifications (H3K4me3; Millipore, 07–473; H3K9me3, Abcam, ab8898; H3K27me3, Millipore, 07–449), DNA modifications (5-hydroxymethylcytosine and 5-methylcytosine) and collagen markers (collagen-II and collagen-X). Details on antibodies and precedures are shown in Supplementary Table 1.

### Bone development in zebrafish embryos

After fertilization (*d* = 0) embryos were positioned on a 10 cm Petri dish coated with 1% agarose and injected with 1 nl 0.8 M D-2-HG, L-2-HG or PBS during the 2–8 cell stages of development [41]. The compounds were loaded into borosilicate glass capillary needles (1.0 mm OD × 0.78 mm ID × 100 mm L; Harvard Apparatus) and the injections were performed using a Pneumatic Pico pump. Per experiment approximately 20 zebrafish embryos per group were used. Experiments with PBS and D-2-HG conditions were performed twice at different time points. At 8 days post fertilization (dpf) water was removed and 4% paraformaldehyde was added to fix the zebrafish embryos. Double staining with alizarin red and alcian blue was performed as described elsewhere [42]. Alizarin Red positive vertebrate rings were counted to quantify bone development. To determine statistical significance an independent sample *t*-test was performed using SPSS v20.

## SUPPLEMENTARY FIGURES AND TABLES


