# Supplementary material for: The oncometabolite D-2-hydroxyglutarate induced by mutant IDH1 or -2 blocks osteoblast differentiation in vitro and in vivo
Source: Oncotarget. 2015 May 25;6(17):14832–42. doi: 10.18632/oncotarget.4024 (PMC4558118; doi:10.18632/oncotarget.4024)
Supplement: Supplementary file 1 [file oncotarget-06-14832-s001.pdf]

SUPPLEMENTARY FIGURES AND TABLES

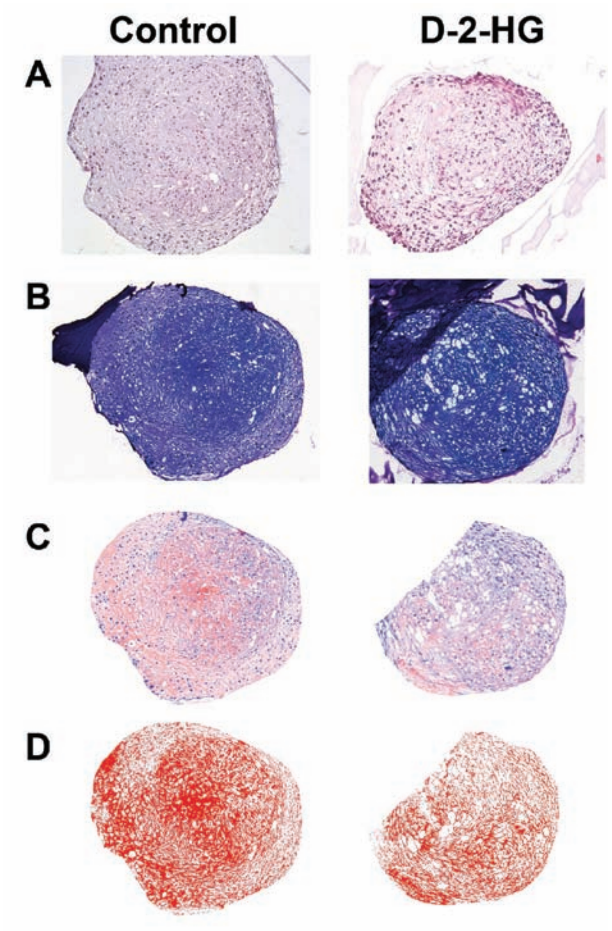

Supplementary Figure S1: Step-by-step quantification of metachromasia in toluidine blue staining after chondrogenic differentiation. A. H&E staining of pellets; **B.** toluidine blue staining of pellets; **C.** images of toluidine blue stained pellets after spectral imaging; **D.** Unmixing of spectra purple stained regions are shown. Quantification of metachromasia in toluidine blue staining after chondrogenic differentiation was performed on the unmixed pictures. Using ImageJ the percentage of pellets stained was determined.

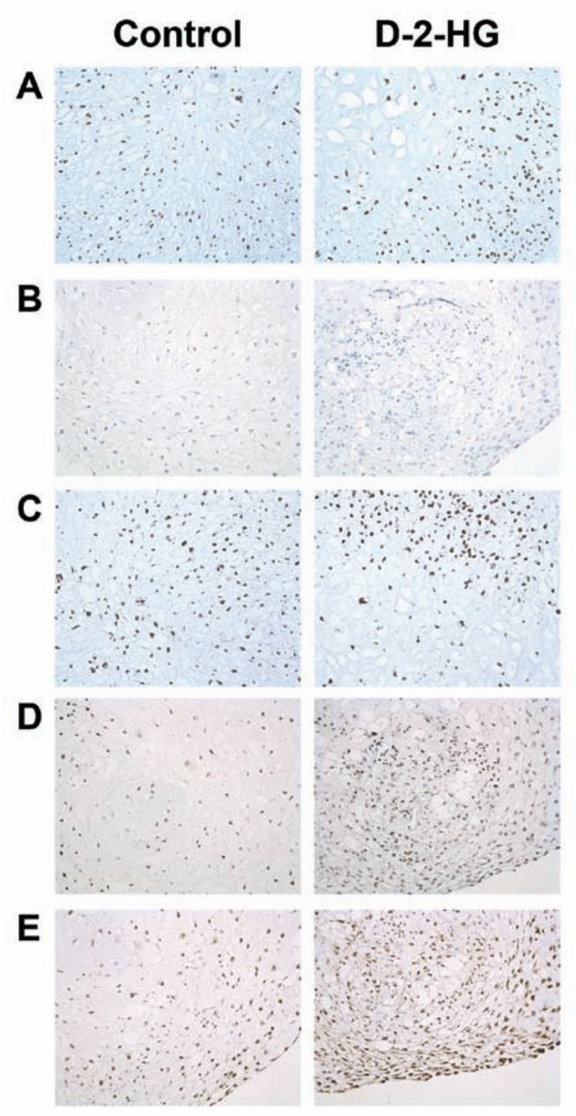

Supplementary Figure S2: Staining of pellets for histone modification markers (trimethylation of H3K4, H3K9 and H3K27) and DNA methylation markers (5-methylcytosine and 5-hydroxymethylcytosine). Staining of pellets for H3K4me3 **A.** H3K9me3 **B.** H3K27me3 **C.** 5-methylcytosine **D.** and 5-hydroxymethylcytosine **E.** did show high basal levels in the controls, therefore no difference could be observed between samples treated with 5 mM D-2-HG and controls.

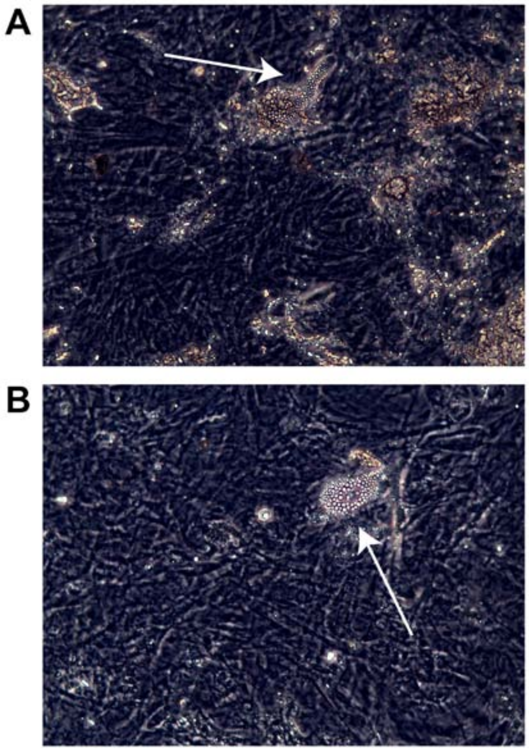

Supplementary Figure S3: Pictures of wells with MSCs allowed to differentiate under osteogenic differentiation conditions. A. MSC allowed to differentiate under osteogenic differentiation conditions treated with PBS. Cells show calcification as well as spontaneous adipocyte differentiation (white arrow). **B.** allowed to differentiate under osteogenic differentiation conditions treated with 5 mM D-2-HG. Cells still show spontaneous adipocyte differentiation (white arrow), whereas calcification is blocked.

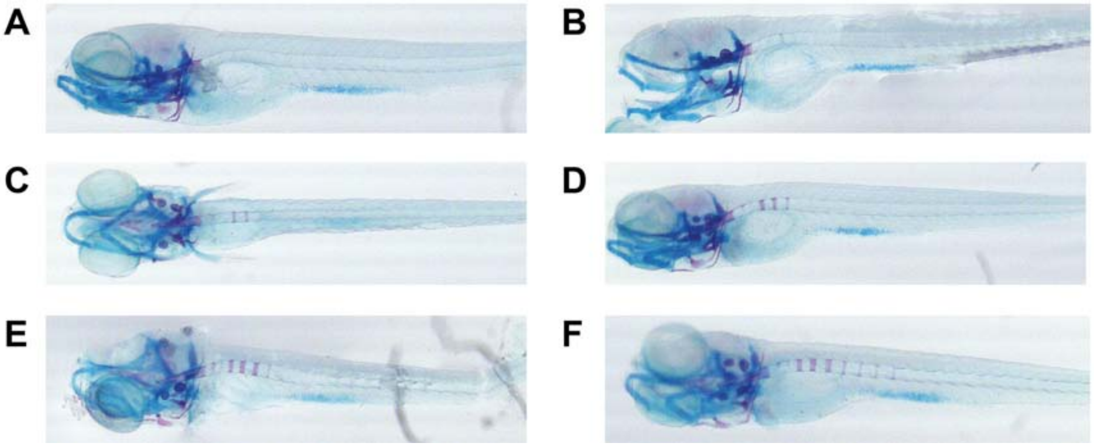

Supplementary Figure S4: Quantification of osteoblast differentiation in zebrafish by counting the Alizarin Red positive vertebrate rings. Double staining of zebrafish 8 days after fertilization with Alcian Blue and Alizarin Red. Zebrafish shows 0 **A.** 1 **B.** 2 **C.** 3 **D.** 4 **E.** up to 7 **F.** Alizarin Red positive vertebrate rings. Number of Alizarin red positive vertebrate rings were used as a measure of skeletal development.

Supplementary Table S1. Specifications of antibodies used for immunohistochemistry

Antibody	Company	Clone	Antigen retrieval	dilution	IHC remarks
Col X	Quartett		protK + hyaluronidase	1:100	
Col-II	Neomarkers	2B1.5	protK + hyaluronidase	1:100	
H3K27-me3	Millipore		Tris-EDTA	1:7000	block 30’ in Elk
H3K4-me3	Millipore		Tris-EDTA	1:7000	block 30’ in Elk
H3K9-me3	Abcam		Tris-EDTA	1:2000	block 30’ in Elk, 1st ab in Elk
5-hmC	Active motif		Citrate	1:7000	block 30’ in Elk
5-mC	Millipore	33D3	Tris-EDTA	1:2000	block 30’ in Elk
